# Pneumothorax in Patients with Pulmonary Langerhans Cell Histiocytosis

**DOI:** 10.1007/s00408-018-0155-1

**Published:** 2018-09-05

**Authors:** E. Radzikowska, K. Błasińska–Przerwa, E. Wiatr, I. Bestry, R. Langfort, K. Roszkowski-Śliż

**Affiliations:** 1 III Department of Lung Diseases, National Tuberculosis and Lung Diseases Research Institute, Plocka 26 st., 01-138 Warsaw, Poland; 2 Radiology Department, National Tuberculosis and Lung Diseases Research Institute, Warsaw, Poland; 3 Pathology Department, National Tuberculosis and Lung Diseases Research Institute, Warsaw, Poland

**Keywords:** Histiocytosis, PLCH, Pulmonary function test, Computed tomography

## Abstract

**Introduction:**

Pneumothorax often develops in pulmonary Langerhans cell histiocytosis (PLCH), but some patients take a long time to be correctly diagnosed.

**Objectives:**

This study assessed the frequency of pneumothorax in PLCH and analysed the role of chest computed tomography (CT) in the prompt diagnosis.

**Patients and material:**

Of the 90 patients with PLCH seen from 2000 to 2015, 29 (32%) had pneumothorax as the initial finding. In this group, 18 (62%) patients were diagnosed within 1 month, whereas the diagnosis was delayed for 4–120 months in 11 (38%) patients.

**Results:**

Patients who had pneumothorax as the initial sign of PLCH tended to be younger (mean age 27.7 ± 7.92 vs. 39.9 ± 13.21 years; *P* = 0.0001), male (69% vs. 43%; *P* = 0.028), smoked less (mean pack/years 8.4 ± 6.85 vs. 19 ± 17.16; *P* = 0.003), and had a significantly lower mean FVC (77.96 ± 19.62 vs. 89.47 ± 21.86% pred.; *P* = 0.015) and FEV_1_ (68.6 ± 19.93 vs. 79.4 ± 21.48% pred.; *P* = 0.03 than patients who had no pneumothorax. Recurrent pneumothorax was diagnosed more frequently in the group with a delayed diagnosis (82% vs. 39%; *P* = 0.02). CT was performed in all of the patients who were diagnosed promptly, but in none of the patients with a delayed diagnosis.

**Conclusions:**

Patients who had pneumothorax as the initial sign of PLCH were younger, more frequently men, and had greater respiratory impairment than those who had no pneumothorax. CT in patients with pneumothorax led to a correct diagnosis of this disease.

## Introduction

Langerhans cell histiocytosis (LCH) is a rare disease caused by the proliferation of abnormal bone marrow-derived, CD1a-positive Langerhans cells, which infiltrate various organs [1, 2]. LCH lesions can be observed in multiple organs or a single organ, usually the skin, bone, lymph nodes, or lungs. Pulmonary Langerhans cell histiocytosis (PLCH) is a smoking-related disease characterised by the development of nodular and cystic lung lesions that predispose to pneumothorax [3–7]. It is a disease of young and middle-aged people, mainly smokers, and affects both sexes equally. The course of LCH is unpredictable, and ranges from spontaneous regression (particularly in adults with pulmonary involvement who quit smoking) and stabilisation with or without relapses to continuous progression despite aggressive chemotherapy [3–7].

Pneumothorax often develops in PLCH and is very suggestive of the disease [8, 9]. Nevertheless, some patients are not diagnosed correctly or take a long time to be diagnosed. In PLCH, smoking cessation is the most important recommendation, and the value of a prompt diagnosis cannot be overestimated.

This study assessed the frequency of pneumothorax in PLCH and analysed these patients, focusing on delayed diagnosis and the role of high-resolution computed tomography (HRCT) in the prompt diagnosis of PLCH.

## Patients and Methods

From 01 January 2000 to 31 December 2014, data on 90 patients with PLCH were collected from our database. Multisystem LCH was diagnosed in 20 (22%) patients and isolated pulmonary disease was diagnosed in 70 (78%) patients. Seventy-nine patients were diagnosed based on clinical and radiological findings and were confirmed by histological assessments of lung (70 cases) or bone (9 cases). In six patients, the diagnosis was established by the presence of more than 5% CD1a-positive cells in bronchoalveolar lavage fluid, and in five patients, diagnosis was based on the characteristic clinical and radiological findings and the exclusion of other possible causes of pulmonary lesions. Twenty-nine (32%) patients had pneumothorax as an initial sign of pulmonary lesions before diagnosis. Data were gathered on patient sex, age, smoking history, symptoms, episodes of pneumothorax, results of pulmonary function tests (PFTs), computed tomography (CT), other organ involvement, histological examination of samples, date of first symptom(s), date of diagnosis, and date of last visit. For patients with pneumothorax as a first sign of PLCH, pulmonary function tests (PFTs) were performed according to the joint guidelines of the American Thoracic Society and European Respiratory Society 3–6 months after the episode. The lung volumes were measured using body plethysmography (Jaeger MasterScreen software ver. 4.65; Würzburg; Germany) and the diffusion capacity of the lungs for carbon monoxide (DL_CO_) was determined using the single-breath technique. The predicted values were analysed.

Of patients with pneumothorax, 18 (62%) were diagnosed within 1 month and made up the no-delay (ND) group, while the diagnosis was delayed for between 4 and 120 (mean 37.64 ± 41.79) months in 11 (38%) patients who made up the delayed (D) group.

First and second episodes of pneumothorax were treated by chest tube drainage, however, in some recurrent cases with pleurodesis. The persistent air leak and multiple pneumothoraces were indication for videoassisted thoracoscopy with pleurodesis.

### Statistical Methods

The statistical analyses were performed using Statistica 10 (StatSoft, Tulsa, OK, USA). Continuous variables were compared using an unpaired Student’s *t* test or the Mann–Whitney *U* test. Fisher’s exact test and the χ^2^ test were used to assess proportions. *P* values less than 0.05 were considered statistically significant. All *P* values were two-sided and unadjusted for multiple testing.

## Results

Table 1 summarises the characteristics of the study groups. One-third of patients with PLCH experienced spontaneous pneumothorax as the initial presentation of the disease. One patient initially had a bilateral pneumothorax. There were no significant differences in the distribution of patients with multisystem LCH between the two groups. Men (69% vs. 43%; *P* = 0.028) and young patients (mean age 27.7 ± 7.92 vs. 39.9 ± 13.21 years; *P* = 0.0001) were overrepresented in the group of patients with pneumothorax as the first sign of PLCH. Only 2 of the 90 patients were non-smokers: a 15-year-old boy with no significant exposure to cigarette smoke and a woman who was passively exposed to cigarette smoke. Patients who had pneumothorax at the presentation of the disease smoked less than those without it as the initial sign of PLCH (mean pack/years, 8.4 ± 6.85 vs. 19 ± 17.16; *P* = 0.003). Pneumothorax recurred in 19 patients (21%), of whom 16 (55%) had pneumothorax as the first sign of PLCH. In one patient with multisystem LCH, recurrent pneumothorax was observed during the course of disease progression. Two other patients also had one recurrent pneumothorax each, also linked with disease progression. Patients who had pneumothorax as the first sign of PLCH had a significantly lower mean forced vital capacity (FVC)(77.96 ± 19.62 vs. 89.47 ± 21.86% of pred.; *P* = 0.015), forced expiratory volume in one second (FEV_1_)(68.6 ± 19.93 vs. 79.4 ± 21.48; *P* = 0.03), and total lung capacity (TLC)(90.6 ± 15.74 vs. 104.9 ± 17.26% pred.; *P* = 0.007) compared to patients who had no pneumothorax. More frequently restrictive pattern of respiratory impairment (TLC < 80% pred.) was noticed in patients with than without pneumothorax (22% vs. 3% ; *P* = 0.005). Only 12% of patients had TLC over than 120% of pred., but in 50% of patients RV over than 120% pred. was shown. There were no differences in distribution of patients with symptoms of hyperinflation between patients who had pneumothorax as a initial sign of the PLCH or this without this symptom. The PLCH diagnosis in patients with pneumothorax was established more frequently during the first 6 months after this episode than in those without it (45% vs. 21%; *P* = 0.036) (Table 1). During the time of observation, patients who had pneumothorax as the initial sign had more frequently recurrent episodes of this condition, than those who did not have pneumothorax at presentation (55% vs. 5%; *P* < 0.001).

**Table 1:** Patients characteristics

	All	Patients with pneumothorax as the initial sign	Patients without pneumothorax as an initial sign	*P*
No. of patients	90	29 (32%)	61 (68%)	0.028
Women	43 (48%)	9 (31%)	34 (56%)	
Men	47 (52%)	20 (69%)	27 (43%)	
Multisystem				
LCH	20 (22%)	4 (14%)	16 (26%)	0.185
PLCH	70 (78%)	25 (86%)	45 (74%)	
Age (years)	35.78 ± 13.24	27.1 ± 7.92	39.9 ± 13.21	0.001
Smoking Pack/years (mean ± SD)	15.87 ± 15.14	8.4 ± 6.85	19 ± 17.16	0.003
Non-smokers	2	1	1	
Smokers	88	28	60	
Pneumothoraces during the observation period (Mean; range)	0.84 (0–8)	2.41 (1–8)	0.1 (0–4)	0.0001
Number of patients with recurrent pneumothoraces	19 (21%)	16 (55%)	3 (5%)	< 0.0001
Time to diagnosis months (mean ± SD)	16.87 ± 28	20.93 ± 37.5	15.01 ± 22	0.276
Observation time months (mean ± SD)	62.9 ± 48.61	68.2 ± 56.96	60.9 ± 65.22	NS
Number of patients with diagnosis delay > 12 months	33 (37%)	11 (38%)	22 (36%)	0.5
Number of patients with diagnosis delay < 6 months	26 (29%)	13 (45%)	13 (21%)	0.036
FVC% pred (mean ± SD)	85.79 ± 21.38	77.96 ± 19.62	89.47 ± 21.86	0.015
FEV_1_% pred (mean ± SD)	76.06 ± 21.38	68.6 ± 19.93	79.4 ± 21.48	0.03
FEV_1_%VC < 70% pred	19 (22%)	6 (22%)	13 (22%)	0.95
DL_CO_ % pred (mean ± SD)	59.22 ± 15.62	60.8 ± 11.31	58.5 ± 17.4	0.88
TLC% pred (mean ± SD)	100.2 ± 17.83	90.6 ± 15.74	104.9 ± 17.26	0.007
TLC > 120% pred	10 (12%)	2 (7%)	8 (16%)	0.41
TLC < 80% pred	8 (9%)	6 (22%)	2 (3%)	0.005
RV% pred (mean ± SD)	128.55 ± 42.2	126.15 ± 25.91	129.66 ± 48.31	0.36
RV > 120% pred	44 (51%)	14 (52%)	30 (51%)	0.93
RV < 80% pred	5 (6%)	1 (4%)	4 (7%)	NS
PaO_2_ mmHg (mean ± SD)	78.2 ± 9.48	80.77 ± 9.22	77 ± 9.52	0.89

*FVC* forced vital capacity, *FEV1* forced expiratory volume in 1 s, *TLC* total lung capacity, *RV* residual volume, *DL*_*CO*_ diffusion lung capacity for carbon monoxide, *PaO*_2_ partial pressure of oxygen in the arterialized blood, *LCH*, Langerhans cell histiocytosis, *PLCH* pulmonary Langerhans cell histiocytosis, *pred*. predicted, *SD* standard deviation

Persistent air leakage resulted in subsequent surgical procedures in four patients (14%) in the ND group (*P* = 0.007). Recurrent pneumothoraces were more frequent in the D group than in the ND group (82% vs. 39%; *P* = 0.02) and were observed 1–72 (mean 32 ± 28.82) months after the first episode. In addition, patients with a delayed diagnosis experienced more recurrences than those with a prompt diagnosis (3.45 vs. 1.78; *P* = 0.019; Table 2). CT was performed at the time of first presentation of pneumothorax in all of the patients in the ND group, but in no case in the D group (*P* = 0.0009). An incorrect initial diagnosis caused very long delays in diagnosis in three patients: two men without pneumothorax (diagnosis delay 120 months) were originally diagnosed with hypersensitivity pneumonitis and autoimmune hepatitis, and one woman with pneumothorax as the initial sign (diagnosis delay 176 months) was diagnosed with lymphangioleiomyomatosis. Patients with pneumothorax with a delayed diagnosis had similar pulmonary function as those with a prompt diagnosis (Table 3).

**Table 2:** Characteristics of the patients with pneumothorax

	Patients with a prompt diagnosis (ND)	Patients with a delayed diagnosis (D)	*P*
Patients (number)	18	11	0.73
Women	6	3	
Men	12	8	
Age (years, mean ± SD)	25.6 ± 8.02	29.55 ± 7.46	0.15
Smoking (pack/years, mean ± SD)	6.22 ± 6.89	11.09 ± 5.95	0.026
Number of patients with multiple pneumothoraces	7 (39%)	9 (82%)	0.02
Mean number of pneumothoraces (range)	1.78 (1–6)	3.45 (1–8)	0.019
Dyspnoea	10 (56%)	9 (82%)	0.15
Weakness	11 (61%)	11 (100%)	0.5
Chest pain	8 (44%)	5 (45%)	0.7
Sweat	1 (6%)	1 (9%)	NS
Fever	1 (6%)	0	NS
Weight lose	5 (28%)	6 (55%)	0.15
Cough	13 (72%)	11 (100%)	0.036

*SD* standard deviation, *NS* non-significant

**Table 3:** Pulmonary function tests in PLCH patients with pneumothorax

	Patients without delay (ND)		Patients with delay (D)		*P*
	No		No		
FVC% pred. (mean ± SD)	16	77.26 ± 20.78	11	78.9 ± 18.86	0.59
FEV_1%_ pred. (mean ± SD)	16	68.86 ± 19.96	11	66.63 ± 20.07	0.79
FEV_1_% FVC (mean ± SD)	16	87.26 ± 17.38	11	79.0 ± 14.5	0.16
FEV_1_% FVC < 70% pred	16	6	11	0	NS
DL_CO%_ pred. (mean ± SD)	16	62.46 ± 13.44	11	58.54 ± 7.54	0.36
TLC% pred. (mean ± SD)	16	90.4 ± 16.27	11	90.81 ± 15.78	0.97
TLC > 120% pred	16	1	11	1	NS
RV% pred. (mean ± SD)	16	122.53 ± 28.9	11	131.09 ± 21.5	0.35
PaO_2_ mmHg (mean ± SD)	17	83.44 ± 8.69	11	76.9 ± 8.93	0.07

*FVC* forced vital capacity, *FEV1* forced expiratory volume in 1 s, *TLC* total lung capacity, *RV* residual volume, *DL*_*CO*_ diffusion lung capacity for carbon monoxide, *PaO*_2_ partial pressure of oxygen in the arterialized blood, *pred*. predicted, *SD* standard deviation

In observed group of patients, 75 (70 in patients with pneumothorax as an initial sign, and five in patients without pneumothorax at the beginning of the disease) episodes of pneumothorax (42 on the left side, and 31 on the right side) were noticed. Eight patients had bilateral recurrent pneumothoraces. Seventy episodes of pneumothorax were treated with chest tube drainage, and 5 (6%) by posturnal drainage. Ten patients (14%) had only one episode of pneumothorax, successfully treated with pleural drainage. Persistent air lick, in spite of pleural drainage was shown 10 (14%) times. Recurrent pneumothorax after the chest tube drainage was noticed 50 (71%) times. Mechanical pleurodesis were performed 7 (10%) times, and chemical pleurodesis 3 (4%) times. 37 (53%) episodes of recurrent pneumothorax were noticed in a month, 6 (9%) in a period of 2–3 months, and 4 (7%) after 1 year. Reactivation of the disease after 2, and after 7 years was connected with pneumothorax in one patient and in two patients, respectively. Ipsilateral recurrence of pneumothorax was noticed 33 (66%) times and recurrence to the contralateral side 17 (34%) times. Recurrent pneumothoraces in a month and in other case after 4 months from the mechanical pleurodesis were observed. Chemical pleurodesis was ineffective in one patient, and recurrence of pneumothorax was observed after a month from this procedure. Seven years after the mechanical and chemical pleurodesis in the course of PLCH progression pneumothorax was observed in two patients. Recurrent pneumothoraces were observed more frequently in patients with multisystem disease, than in those with isolated PLCH (45% vs. 15% ; *P* = 0.005).

Patients were under the observation for a mean time of 62.9 ± 48.61 months, and three were no significant differences between groups in the time of follow-up (Table 1).

## Discussion

We found that patients who had pneumothorax as a first sign of PLCH were younger, more frequently men, smoked fewer cigarettes, and had greater respiratory impairment than patients who did not have pneumothorax at presentation.

Pneumothorax is a very important and suggestive sign of cystic lung disease [10–12]. Pneumothorax caused by PLCH accounts for 0.25–0.5% of all spontaneous pneumothoraces each year in the United States [20]. The frequency of patients who have pneumothorax as the initial sign of PLCH ranges widely from 10 to 32% in different patient cohorts [4, 6, 9, 12–15]. As in Mendez et al., our patients with PLCH who experienced pneumothorax were more frequently younger men; however, Mendez et al. did not observe more severe respiratory impairment in patients with pneumothorax, contrary to the patients in our study [9]. About 20% of the our PLCH patients experienced recurrent pneumothoraces, which were more frequent in patients who had pneumothorax at the beginning of the disease, which potentially had a negative influence on pulmonary function at the time of presentation. A multicentre, prospective, observational study of 58 PLCH patients conducted in France found that 19% of patients had history of pneumothorax and two patients who experienced pneumothorax during follow-up [12]. Additionally, it was shown that smoking status and PaO_2_ were risk factors for lung function deterioration during the first years after diagnosis. This study proved that smoking cessation is the most important recommendation for PLCH patients [12]. Therefore, a prompt diagnosis is very important; however, less than 50% of patients with PLCH give up this addiction, in spite of diagnosis [16–19]. About 70% of our patients stated that they had quit smoking, but this was not proven objectively.

All of our patients who underwent HRCT at the time of first pneumothorax were diagnosed correctly. No patients who were diagnosed more than 12 months after pneumothorax underwent HRCT. A routine chest X-ray has very limited sensitivity and specificity for detecting cystic lesions in the lungs compared to CT. Recently, Gupta et al. showed that HRCT is cost-effective for screening for rare cystic lung diseases, such as Birt–Hogg–Dubé syndrome, LCH, and lymphangioleiomyomatosis, in patients with spontaneous pneumothorax, despite the fact that the prevalence of these diseases is lower than 0.01% [20].

We believe that CT should be used to assess patients with spontaneous pneumothorax, as it is not only very sensitive, but is also cost-effective for diagnosing rare cystic lung diseases. Experience with low-dose CT in patients with pulmonary nodules suggests that this examination can be used with other indications, including multicystic lung diseases. Low-dose CT has a lower sensitivity for detecting interstitial lesions than standard-dose CT, but the exposure dose is significantly lower (1.5 vs. 7 msv) [21, 22]. Therefore, we suggest the recommendation of the low-dose chest CT in all patients with spontaneous pneumothorax.

Our patients were mainly treated with chest tube drainage, which was successful in distance of a year in 14% of cases. In a period of 1 year, mechanical pleurodesis was effective in 71% patients and chemical in 66% patients, but in a long observation, additional recurrent pneumothoraces were noticed. On contrary, Mendez et al reported that surgical management of pneumothorax was very effective, and no case of recurrent pneumothorax was noticed after pleurodesis [9]. In our patients, recurrent pneumothorax occurred mainly at the beginning of the disease (during the first month), in active phase of PLCH or in the time of relapse. In addition, patients with multisystem rather than isolated PLCH had recurrent pneumothoraces more frequently (45% vs.15% ; *P* = 0.005). This finding also supports link between active disease and pneumothorax. There were no patients who had mechanical or chemical pleurodesis during the management of the first episode of pneumothorax. Those patients were treated mainly in district hospital, on usual surgery departments, in which chest tube drainage was preferred. Due to high rate of recurrence, pleurodesis is advised following the initial episode of pneumothorax, but before patients should be diagnosed. Regarding this recommendation, CT scan in patients with pneumothorax should be performed at the initial episode of pneumothorax [20].

There were some limitations to this study. It was a retrospective, single-centre study. The presented pulmonary function parameters were the first measurements performed in our hospital. The patients were referred after different lengths of time from the first sign of PLCH, or even from diagnosis, therefore it might influence on results of pulmonary function. In patients who had a pneumothorax as the first sign of the disease, the PFTs were done 3–6 months after this episode. Lower values of pulmonary function test in patients, who had a history of pneumothorax might be connected with pleural lesions caused by pleural derange, pleurodesis, or pleurectomy. From the other side, more than 50% of patients who did not have pneumothorax as an initial symptom had videoassisted thoracoscopy (usually in regional hospitals) for PLCH diagnosis, and it might also influence slightly lower PFTs parameters in those patients, measured in the time of first presentation in our hospital.

Nevertheless, the focus of this study was to underline the role of chest CT in the rapid, correct diagnosis of PLCH, because it is translated into better outcomes for these young patients.

## Conclusion

Patients who had pneumothorax as the first sign of PLCH were more frequently men, younger, smoked more intensively, experienced recurrent pneumothoraces, and had greater respiratory impairment than patients who had no pneumothorax. Chest CT in patients with pneumothorax led to a prompt diagnosis of PLCH and CT should be performed in all young patients with spontaneous pneumothorax. Recurrent pneumothorax is a symptom of active disease.

## Abbreviations

CT: Computed tomography
DL_CO_: Diffusion lung capacity for carbon monoxide
FEV_1_: Forced expiratory volume in 1 s
FVC: Forced vital capacity
LCH: Langerhans cell histiocytosis
MS LCH: Multisystem Langerhans cell histiocytosis
PaO_2_: Partial pressure of oxygen in the blood
PLCH: Pulmonary Langerhans cell histiocytosis
Pred.: Predicted
RV: Residual volume
TLC: Total lung capacity
